# Pediatric pain knowledge and attitudes among health care professionals—A National Danish Survey

**DOI:** 10.1002/pne2.12104

**Published:** 2023-04-10

**Authors:** Gitte Würtz, Christina Schmidt, Claus Sixtus Jensen, Grete Teilman, Hanne Konradsen

**Affiliations:** ^1^ Department of Pediatrics Herlev and Gentofte University Hospital Herlev Denmark; ^2^ Department of Pediatrics and Adolescent Medicine Aarhus University Hospital Aarhus Denmark; ^3^ Research Center for Emergency Medicine Aarhus University Hospital Aarhus Denmark; ^4^ Department of Clinical Medicine Health Aarhus University Aarhus Denmark; ^5^ Department of Pediatrics and Adolescent Medicine, Nordsjællands Hospital University of Copenhagen Hillerød Denmark; ^6^ Department of Gastroenterology Herlev and Gentofte University Hospital Herlev Denmark; ^7^ Division of Nursing, Department of Neurobiology, Care Science and Society Karolinska Institutet Stockholm Sweden; ^8^ Department of Clinical Medicine, Faculty of Health and Medical Sciences University of Copenhagen Copenhagen Denmark

**Keywords:** collaboration, health service, knowledge, pain, pain management

## Abstract

**Aim:**

To explore and compare Danish health‐care professionals’ attitudes and knowledge towards pain management of children.

**Methods:**

The cross‐sectional study was carried out using the Pediatric Pain Knowledge and Attitudes Questionnaire. The questionnaire was distributed to all health care professionals caring for children in three hospital settings, including nurses and physicians in departments of pediatric, emergency, and anesthesia and medical laboratory technologists.

**Results:**

The study was conducted in 2020 and 765 health care professionals participated. Within the six main categories included in the questionnaire, there were significant differences between nurses and physicians in three subcategories: view on the care of children in pain, using drugs to relieve pain, and the four mandatories. Comparing nurses with medical laboratory technicians, there were significant differences in the subcategory "view on the care of children in pain." Comparing types of clinical departments, there were significant differences in the subcategories’ view on the care of children in pain, using drugs to relieve pain, and the four mandatories. Overall, we found that the participating health professionals did not have a uniform understanding of pain management and therefore might treat children differently.

**Conclusion:**

The present study highlights the need to align health care professionals’ knowledge regarding pain assessment and management of children, as well as the need to develop and test interventions that support the use of knowledge in practice.

## 1 INTRODUCTION

Pain management in children admitted to hospitals has been inadequate in many countries for decades.^1^, ^2^, ^3^ Procedural pain, such as blood sampling, is one of the most commonly reported forms of pain in children,^1^, ^4^ but children may experience a range of up to 20 painful procedures per day when hospitalized.^5^ Interviews with children who experienced pain in hospital settings showed that they did not think their pain was adequately managed.^1^, ^6^ Hospitalized children have also described how pain increased when they were in unknown situations, increasing even more when various health care professionals such as physicians, nurses, and medical laboratory technologists (MLT) performed painful procedures.^1^, ^6^ Children admitted to hospitals in acute situations or with chronic health problems have been reporting their experiences of not feeling consulted about their pain experiences, resulting in the feeling that their concerns are not being taken into account by health care professionals and their parents.^7^, ^8^ As an example, a study of health professionals' views on physically restraining children during clinical procedures^9^ found that various factors such as time and “getting the procedure over and done” undermined the child's expressed wishes against being held.

Many factors can influence pain management in a hospital, including the level of knowledge and experience of caring for children, negative attitudes towards the use of opioids among health care professionals and organizations, and the use of guidelines or standards.^10^ Studies have pointed towards health care professionals' lack of theoretical knowledge concerning children's perception of pain during admissions, which plays a vital role in how health‐care providers manage pain for this group of patients.^11^, ^12^ Furthermore, health care professionals do not necessarily use the theoretical knowledge they have during treatment and care,^11^, ^13^ and their attitudes towards pain assessment and treatment may influence children's experience of pain. Health care professionals often rely on children's nonverbal behaviors rather than their self‐reported experience of pain when deciding on whether to treat the pain.,^13^ this might lead to children and adolescents feeling disregarded.^14^

Some evidence exists of health care professionals' knowledge of the care of children in pain. In a study of nurses' knowledge and attitudes in pediatric postoperative pain management, it was found that although nurses had knowledge of pediatric pain management, they did not always use this knowledge.^15^ Nurses and physicians have general and basic knowledge on pain management, but often miss specific knowledge on how to care for children in pain.^16^ Likewise, graduating students have also been shown to lack knowledge on pain management of children.^17^

Hospitalized children encounter a variety of health care professionals, including MLT, intensive care unit nurses, residents, pediatric residents, nurses, and physicians from different surgical specialities and the emergency department (ED). The different groups of health care professionals may care for children in pain at one point or another, and therefore knowledge and understanding of children's pain experiences is crucial. Thus, it is important to gain insight into knowledge and attitudes towards children's pain management in different groups of health care professionals that interact with children during their hospitalization.

This study aimed to explore and compare health care professionals' attitudes and knowledge towards pain management of children in Denmark.

## 2 METHOD

### 2.1 Design

A cross‐sectional design was chosen for this study.

### 2.2 Data

The Pediatric Pain Knowledge and Attitudes Questionnaire (PPKAQ) was used for data collection. The PPKAQ was initially developed in Finland in 1999^18^ and underwent an English validation in 2010.^19^ Before collecting data in Denmark, a Danish version of the questionnaire underwent validation and reliability testing.^20^ The reliability test showed a medium‐to‐high Cronbach's alpha with a range from 0.804 to 0.906, and intrarater reliability measured by Spearman correlations (rho), ranging from 0.683 to 0.83. Included in the Danish version was an additional category containing 14 questions linked to recommendations from the Danish information centre on children and pain.^21^ The recommendations include “the four mandatories”: using local anesthesia, giving oral sucrose or breastfeeding, using comfort positioning, and using age‐appropriate distraction.^21^, ^22^ The addition was made to include up‐to‐date knowledge on pain treatment within hospitalized children.

The Danish version of PPKAQ then consisted of 83 questions. All items were scored on a 5‐point Likert scale from 1 = agree to 5 = disagree. An extra response category was added with the option “do not know.” This category was added to the questionnaire, as members of additional health care professions were included compared to the original study, and because data from the Danish validation showed that not all professions were trained in all categories.^20^ The questionnaire is testing differences in attitudes, and it is not possible to transfer a low or a high score to a correct answer or detect a clinically important difference. Items were summarized in each of the six subscales: A, *views on the care of children in pain*; B, *physiology of pain*; C, *nondrug methods of pain relief*; D, *using drugs to relieve pain*; E, *sociology and psychology of pain*; and F, *the four mandatories*.

The following demographic data were also collected: age, gender, health care profession, and type of clinical department the participant was employed in.

### 2.3 Sample and setting

The survey was conducted from November 2019 to January 2020. Three hospitals participated, of which two were in the capital region of Denmark and one in the Central Denmark Region. All clinical personnel who cared for children were approached by email.

The following group of health care professionals were approached:

1. Nurses employed in the following departments: pediatrics, emergency, anesthesia, and intensive care.
2. Physicians employed in the following departments: pediatrics, anesthesia, orthopedic surgery, and otorhinolaryngology.
3. Medical laboratory technologists.

In the survey invitation, it was written that if the person were not involved in care for children, they should please ignore the survey. The questionnaire was sent out a second time 3 weeks later to those who did not respond in the first round.

### 2.4 Statistics

Data were transferred into SPSS IBM 25 (IBM, 2017). Items in the PPKAQ were grouped according to subscales and analyzed using descriptive statistics. Before comparing health care professions and types of departments, the answering category “do not know” was removed. A one‐way analysis of variance was used to determine whether significant variation existed among subgroups. If a statement was answered by fewer than five participants, it was not included in the analysis.

The physicians employed in surgical departments in a variety of specialties. In total, 39 orthopedic surgeons, 20 abdominal surgeons, 26 otorhinolaryngologic surgeons, and two from urinary tract surgery. A subanalysis comparing their answers showed homogeneity in all subscales. Therefore, data from these groups were merged.

Scores for each main category were presented with a mean score, and differences between *p* values ≤0.05 were considered significant. For the statistical analysis, a statistician was consulted.

Three of the authors thoroughly examined and individually chose five statements in each category they found to be of most clinical relevance, discussed these during an online meeting, and agreed on the final group of questions. These questions are shown in Table 4, where clinical departments are compared. The total list of items is listed in a Appendix S1.

### 2.5 Ethical considerations

This study complied with the principles of the Helsinki Declaration.^23^ As participation in this study was anonymous and only contained data collected from health care professionals, no formal ethical approval was needed according to Danish law. The study was approved by the Danish Data Protection Agency (number P‐2019‐265). The local administrators in each hospital approved that the research group contacted their employees.

## 3 RESULTS

### 3.1 Participants

Seven hundred and sixty‐five health care professionals responded to the survey, which corresponded to a response rate of 40%. Demographics of participants are shown in Table 1.

**TABLE 1:** Participant demographics.

	*n* (%)
Total number	765
Gender	
Male	126 (16.5)
Female	639 (83.5)
Years of work	
0–10	269 (25.1)
>10	496 (74.9)
Profession	
Physician	203 (26.5)
Nurse	464 (60.7)
MLT	70 (9,2)
Not recorded	28 (3.6)
Workplace	
Pediatric department	359 (46)
Anesthesia department	174 (20.4)
Emergency department	65 (8.6)
Biochemical department	77 (9.8)
Surgical department	90 (12)

Abbreviation: MLT, medical laboratory technician.

The scores for all included health care professions and types of medical departments are presented in Table 2. The use of the “do not know” answer was used within each profession throughout the survey but was particularly high within the group of MLT (46%).

**TABLE 2:** Comparison of the mean (SD) score for included health care professionals and type of medical department.

	A) Views on the care of children in pain	B) Physiology of pain	C) Nondrug methods of pain relief	D) Using drugs to relieve pain	E) Sociology and psychology of pain	F) The four mandatories
Professions						
Physicians	54.2 (4.7)^a^ ^,^ ^b^	24.7 (3.8)	21.6 (2.9)	56.3 (4.8)^a^	28.4 (3.9)^a^	41.1(3.8)
Nurses	56.1 (4.2)^b^	25.3 (3.9)	21.3 (3.7)	58.4 (4.1)	30.6 (4.9)	42.3 (4.5)
MLT	46.4 (5.7)	23.7 (2.9)	22.2 (2.8)	No replies	27.4 (5.8)	40.1 (6.9)
Type of ward						
Pediatric ward	55.9 (3.8)	25.1 (3.7)	21.2 (3.3)	57.5 (4.5)	29.8 (6.6)	42.1 (4.4)
Anesthesia	55.5 (4.9)	24.6 (3.6)	21.6 (4.0)	56.9 (4.1)	30.3 (4.5)	42.2 (4.2)
Emergency	54.7 (4.8)	26.8 (2.7)	22.2 (3.3)	58.8 (6.4)	30.2 (5.9)	39.5 (3.5)
Biochemical	47.1 (6.2)^c^	23.2 (2.7)	21.7 (2.9)	58.0 (0.0)	29.4 (6.8)	38.2 (7.1)^c^
Surgery	51.9 (5.7)^c^	24.0 (4.8)	21.4 (3.9)	56.3 (10.2)	28.2 (5.1)	39.0 (4.1)

^a^ *p* value <0.001 in comparison with nurses.

^b^ *p* value <0.001 in comparison with medical laboratory technicians.

^c^ *p* value <0.001 in comparison with pediatric ward.

Abbreviation: MLT, medical laboratory technicians.

### 3.2 Differences between health care professions

No significant differences were found comparing health care professions in the subscales' *physiology of pain*, *nondrug methods of pain relief*, and *the four mandatories*, whereas significant differences were found in the other three subscales.

The subscale*s views on the care of children in pain*, *using drugs to relieve pain*, and *sociology*, *and psychology of pain* differed significantly between health care professions. The significant items from each category are presented in Table 3. In the subscale *views on the care of children in pain*, MLTs and physicians are more inclined to think it is acceptable to draw blood from children without the use of analgesic drugs and that parents exaggerate their child's pain.

**TABLE 3:** Comparing selected items between health care professions.

Item	Health care profession						*p* value		
	Physician		Nurse		MLT		Physician	Physician	Nurse
	*n*	Mean (SD)	*n*	Mean (SD)	*n*	Mean (SD)			
							Nurse	MLT	MLT
*Views on the care of children in pain*									
A1) Children tolerate pain better than adults do	202	4.48 (0.91)	457	4.49 (0.98)	65	3.98 (1.17)	0.837	**0.000**	**0.000**
A3) It is ok to carry out minor procedures, such as taking blood, without using analgesic drugs	202	3.51 (1.45)	462	4.06 (1.30)	71	2.39 (1.42)	**0.000**	**0.000**	**0.000**
A4) Children under two feel less pain than older children in similar situations	195	4.80 (0.69)	438	4.74 (0.74)	55	4.02 (1.38)	0.406	**0.000**	**0.000**
A5) A child who is crying and who says they are experiencing pain is likely to be in pain	202	1.57 (0.73)	453	1.44 (0.71)	66	1.88 (1.10)	**0.040**	**0.004**	**0.000**
A6) Infants who are less than a month old may be intubated without pain medication	163	4.86 (0.52)	358	4.82 (0.68)	16	4.13 (1.31)	0.595	**0.000**	**0.000**
A7) Parents should be involved in the management of their child's fever	203	1.10 (0.32)	459	1.12 (0.41)	66	1.39 (0.72)	0.654	**0.000**	**0.000**
A8) When a child can talk, they should be asked to rate their own pain intensity	200	1.83 (0.95)	447	2.07 (1.18)	61	2.30 (1.14)	**0.011**	**0.004**	0.140
A10) Postoperative pain in children should be eliminated	199	1.02 (0.12)	448	1.01 (0.13)	49	1.37 (0.60)	0.923	**0.000**	**0.000**
A11) When managing chronic pain in children the main goal of treatment is to control the pain	186	1.79 (0.96)	380	1.82 (1.12)	42	1.48 (0.67)	0.744	0.080	**0.044**
A12) Parents exaggerate their child's pain	195	3.51 (1.13)	425	3.96 (1.13)	57	2.74 (1.06)	**0.000**	**0.000**	**0.000**
A13) Children do not need analgesic drugs before having burn dressings changed	178	4.80 (0.60)	400	4.89 (0.49)	31	4.58 (1.06)	0.084	**0.046**	**0.004**
A14) Infants who are less than a month old can be intubated without sedation	157	4.83 (0.56)	320	4.84 (0.60)	13	3.85 (1.57)	0.881	**0.000**	**0.000**
A15) School‐aged children should only be given analgesic drugs if they ask for them	194	3.99 (1.24)	425	4.24 (1.16)	53	3.11 (1.48)	**0.021**	**0.000**	**0.000**
A16) Procedural pain should be eliminated	196	1.21 (0.49)	446	1.20 (0.49)	53	1.81 (0.94)	0.681	**0.000**	**0.000**
A17) The level of pain suffered by a child can be established by giving him placebo medication	148	4.35 (1.07)	317	4.40 (1.00)	24	2.83 (1.09)	0.632	**0.000**	**0.000**
A20) Pain is to be expected if a child is in hospital	192	3.62 (1.33)	436	3.67 (1.36)	57	2.89 (1.42)	0.643	**0.000**	**0.000**
*Using drugs to relieve pain*									
D6) The side effects of nonsteroidal anti‐inflammatory drugs (NSAIDs), for example, diclofenac or ibuprofen, only occur when the drug is given orally	162	4.78 (0.67)	306	4.58 (1.05)	6	4.00 (1.09)	**0.027**	**0.042**	0.381
D12) Nonsteroidal anti‐inflammatory drugs (NSAIDs) can irritate children's digestive systems	168	1.23 (0.62)	332	1.45 (0.81)	14	1.79 (0.80)	**0.002**	**0.008**	0.099
D13) The risk of respiratory depression in children following the administration of opioids is no more likely than in adults, provided that the correct dosage is given	149	1.50 (1.04)	295	1.78 (1.34)			**0.027**		
D24) Nonsteroidal anti‐inflammatory drugs (NSAID) and opioid medication given together provide better analgesic	155	1.34 (0.72)	285	1.56 (0.94)			**0.011**		
*Sociology and psychology of pain*									
E1) The environment in which children grow up has a major influence on the way they express pain	168	1.38 (0.56)	355	1.73 (0.93)	42	1.98 (0.87)	**0.000**	**0.000**	0.072
E2) School‐aged children cannot learn how to use a patient‐controlled analgesia pump	139	4.66 (0.76)	272	4.54 (0.85)	42	4.00 (0.93)	0.168	**0.003**	**0.013**
E3) A child's cultural background affects the way they experience pain	167	1.77 (1.13)	358	1.99 (1.20)	42	2.14 (1.16)	**0.042**	0.065	0.432
E4) The way that children express pain is affected by their temperament	172	1.35 (0.59)	360	1.65 (0.83)	50	1.70 (0.79)	**0.000**	**0.006**	0.653
E5) Changes in a child's behavior can be used to assess their pain	173	1.47 (0.74)	370	1.52 (0.73)	37	1.89 (0.61)	0.474	**0.001**	**0.003**
E6) It is difficult to distinguish between pain and fear in children	173	1.87 (0.85)	370	2.18 (1.03)	50	2.30 (1.09)	**0.001**	**0.006**	0.399
E7) Chronic pain in children does not usually cause mood changes	134	4.69 (0.62)	251	4.48 (0.83)	19	3.53 (1.35)	**0.012**	**0.000**	**0.000**
E8) When assessing a child's pain, it is important to first ascertain their stage of cognitive development	148	2.57 (1.25)	322	3.09 (1.47)	20	2.50 (1.00)	**0.000**	0.839	0.068
E9) The way children experience pain is influenced by their parents' behavior	175	1.31 (0.57)	374	1.55 (0.72)	58	1.40 (0.57)	**0.000**	0.425	0.114
E10) Letting a child know what to expect before a painful procedure will mean that the child experiences less pain than a child not given this information	167	1.58 (0.75)	365	1.61 (0.84)	53	1.40 (0.56)	0.696	**0.006**	**0.006**
E11) Children can sleep even if they are in pain	166	1.99 (1.10)	350	2.31 (1.30)	37	2.32 (1.11)	**0.006**	0.133	0.930
E12) Children between the ages 6–12 months will have no lasting memories of painful procedures	131	4.02 (1.30)	281	4.22 (1.13)	37	2.61 (1.41)	0.102	**0.000**	**0.000**

Abbreviation: MLT, medical laboratory technicians.

In the subscale *using drugs to relieve pain*, only nurses and physicians offered sufficient answers for this study. Exploring single items in this subscale, there was enough statement in two items to compare MLTs. In the items *the side effects of nonsteroidal anti‐inflammatory drugs* (*NSAIDs*), for example, *diclofenac or ibuprofen*, *only occur when the drug is given orally*, and *nonsteroidal anti‐inflammatory drugs* (*NSAIDs*) *can irritate children's digestive systems*. The MLTs differed significantly from the physicians but not the nurses. For the two other statements, *the risk of respiratory depression in children following the administration of opioids is no more likely than that of adults*, *provided that the correct dosage is given* and *nonsteroidal anti‐inflammatory drugs* (*NSAID*) *and opioid medication given together provide better analgesic*, *a* significant difference was found between nurses and physicians.

In the category *sociology and psychology of pain*, significant differences were found across health care professions. All three health care professions disagreed with the assertion that a child's stage of cognitive development is important when assessing its pain.

### 3.3 Differences between departments

We found significant differences between health care professionals in different departments in the subgroups *views on the care of children in pain*, *using drugs to relieve pain*, *sociology and psychology of pain*, and *the four mandatories*. Looking at the data, we found 55 single items in the four categories in which the answers from anesthesia, ED, MLT, and surgery differed significantly from the pediatric department.

The highest number of differences between health care professions from different medical departments were found in the subscale *the four mandatories*. Table 4 shows an excerpt of the answers. The complete table is available in a Appendix S1. Comparing the pediatric department with the biochemical department, 42 items out of 55 were significantly different. Of these items, those related to drug use were the ones that MLTs most often did not answer, and most had used the “do not know” option.

**TABLE 4:** Comparing selected items between medical departments. Pediatric departments used to compare with other departments.

Item	Pediatric		Anesthesia			ED			MLT			Surgery		
	*n*	Mean (SD)	*n*	Mean (SD)	*p*	*n*	Mean (SD)	*p*	*n*	Mean (SD)	*p*	*n*	Mean (SD)	*p*
*Views on the care of children in pain*														
A3) It is ok to carry out minor procedures, such as taking blood, without using analgesic drugs	358	4.06 (1.31)	173	3.97 (1.32)	0.459	65	3.49 (1.48)	**0.002**	78	2.42 (1.44)	**0.000**	89	3.30 (1.48)	**0.000**
A8) When a child can talk, they should be asked to rate their own pain intensity	350	1.99 (1.12)	171	1.82 (1.05)	0.101	62	2.16 (1.24)	0.275	66	2.35 (1.13)	**0.018**	85	2.13 (1.05)	0.314
A10) Postoperative pain in children should be eliminated	352	1.01 (0.07)	169	1.02 (0.13)	0.540	58	1.07 (0.32)	**0.034**	55	1.29 (0.53)	**0.000**	89	1.04 (0.26)	0.116
A16) Procedural pain should be eliminated	348	1.19 (0.50)	168	1.19 (0.46)	0.987	58	1.29 (0.56)	0.181	60	1.85 (0.95)	**0.000**	86	1.22 (0.44)	0.633
A20) Pain is to be expected if a child is in hospital	339	3.65 (1.35)	167	3.74 (1.29)	0.508	58	3.90 (1.32)	0.203	63	2.84 (1.44)	**0.000**	82	3.34 (1.41)	0.062
*Using drugs to relieve pain*														
D1) In treating pain in children only one class of analgesic drug should be used at a time	293	4.65 (0.83)	138	4.67 (0.85)	0.854	47	4.15 (1.25)	**0.001**	17	2.47 (1.07)	**0.000**	60	4.20 (1.39)	**0.001**
D15) Opioid medication given to manage chronic pain in children should be given on a regular basis	192	2.21 (1.37)	113	1.47 (0.82)	**0.000**	23	2.13 (1.36)	0.770	5	2.80 (1.09)	0.279	36	1.81 (1.17)	0.067
D17) Postoperatively children should not be given analgesic drugs until they ask for them	306	4.95 (0.25)	151	4.82 (0.58)	**0.011**	43	4.72 (0.63)	**0.006**	19	4.00 (1.05)	**0.000**	73	4.70 (0.76)	**0.000**
D24) Nonsteroidal anti‐inflammatory drugs (NSAID) and opioid medication given together provide better analgesic	213	1.62 (1.02)	143	1.27 (0.66)	**0.000**	36	1.67 (0.66)	0.762	5	2.20 (1.09)	0.137	58	1.36 (0.64)	**0.044**
D26) Paracetamol and an opioid cannot be given at the same time	283	4.89 (0.44)	145	4.97 (0.34)	0.094	43	4.86 (0.47)	0.624			**0.000**	61	4.92 (0.38)	0.683
*Sociology and psychology of pain*														
E1) The environment in which children grow up has a major influence on the way they express pain	294	1.63 (0.82)	134	1.59 (0.86)	0.628	45	1.73 (0.94)	0.461	45	1.96 (0.93)	**0.018**	63	1.54 (0.88)	0.433
E5) Changes in a child's behavior can be used to assess their pain	306	1.48 (0.70)	143	1.56 (0.78)	0.284	44	1.57 (0.85)	0.454	40	1.85 (0.62)	**0.003**	64	1.42 (0.71)	0.559
E10) Letting a child know what to expect before a painful procedure will mean that the child experiences less pain than a child not given this information	300	1.58 (0.81)	139	1.51 (0.68)	0.415	47	1.83 (1.09)	0.054	58	2.07 (1.01)	**0.000**	61	1.75 (0.77)	0.134
E11) Children can sleep even if they are in pain	296	2.12 (1.21)	133	2.41 (1.31)	**0.028**	44	2.25 (1.31)	0.514	41	2.59 (1.22)	**0.025**	58	2.26 (1.28)	0.434
E12) Children between the ages 6–12 months will have no lasting memories of painful procedures	263	4.24 (1.13)	98	4.30 (1.17)	0.690	27	3.70 (1.10)	**0.027**	35	2.71 (1.46)	**0.000**	35	4.05 (1.27)	**0.003**
*The four mandatories*														
FA19) Restraining small children may have consequences many years ahead	328	1.39 (0.73)	157	1.24 (0.52)	**0.048**	55	1.51 (1.00)	0.310	66	2.15 (1.21)	**0.000**	79	1.68 (0.95)	**0.004**
FC2) The best place for a child to be during a stitching procedure is to sit with a parent	323	1.38 (0.73)	156	1.62 (0.73)	**0.003**	54	1.63 (0.81)	**0.037**	69	2.54 (1.43)	**0.000**	78	1.42 (0.61)	0.662
FC12) If at mother is nervous and scared when her 4‐year‐old child must have a procedure done, it is better if the staff takes over and sends out the parents	312	4.26 (1.08)	154	4.03 (1.23)	**0.049**	52	4.12 (1.32)	0.407	63	2.67 (1.39)	**0.000**	76	3.61 (1.21)	**0.000**
FD2) Local anesthesia (Emla) can be used by all children, even neonate	260	2.93 (1.78)	104	2.12 (1.58)	**0.000**	33	2.33 (1.55)	0.052	43	3.30 (1.63)	0.174	46	1.50 (1.07)	**0.000**
FD9) Relieving pain during a stitching procedure, children in the age of 0–1 year, should be given the opportunity to breastfeed or sugar water	315	1.53 (1.01)	140	1.66 (1.06)	0.215	42	1.64 (1.10)	0.521	64	1.95 (1.16)	**0.003**	60	1.78 (0.94)	0.088

*Note:* Bold values indicate statistically significant results.

Abbreviations: ED, emergency department; MLT, medical laboratory technician.

The results outlined in Table 4 also gave insight into how health care professionals in the pediatric department viewed pain management compared with the surgery department and ED. The health care professionals in these two departments often meet children in acute pain, and it was notable that their answers suggested that they agreed that harmful procedures were done quickly without pain relief.

## 4 DISCUSSION

In this study, we explored different health care professionals' views and attitudes towards caring for children in pain. We found that children are likely to experience several types of pain management when admitted to a hospital, depending on the profession of the health care professional and the department to which they are admitted.

This means that children are unlikely to meet uniform care and treatment, which may have the consequence that they become insecure and unwilling to cooperate.^3^ Studies have shown that pain in children may require additional analgesics or nonpharmacological techniques such as distraction, positioning or feeling of being involved in decisions rather than restraining the patient and “getting the procedure done”.^24^, ^25^ However, in this study, there were significant differences among health professionals' awareness in relation to this aspect of pain management and we also found significant differences dependent on the kind of department. Comparing selected items, significant differences were found such as accepting to carry out minor procedures without analgetic drugs, sending parents out when the procedure is done or waiting to give analgetic drugs until a child asks for it may be considered basic knowledge. Even though specific and advanced knowledge on pain management exists among health care professionals who work in the pediatric setting, basic knowledge must be expected among health care professionals who care for children generally. In this study, significant differences were not found in the subcategories physiology of pain (including questions on the link between acute pain and illness, transmission of pain via the nerve system or the link between pain and increased respiratory rate), nondrug methods of pain relief (including questions on environment and cultural background), and the four mandatories (including using local anesthesia, giving oral sucrose or breastfeeding, using comfort positioning, and using age‐appropriate distraction). The findings in this study may be used to illuminate what knowledge is present across different health care professionals and medical specialties, and what knowledge seems to differ.

Working with children requires increased knowledge about pain, as pain and pain‐related procedures may affect children and their future pain management negatively if not handled properly.^6^, ^7^ Further studies are needed to explore the effect of basic training across medical departments and the effect on children's experiences of pain.

Physicians' and nurses' answers were significantly different within the subcategory *views on pain treatment*. Interviewing children and parents about pain assessment has shown that physicians at the ED significantly underestimate children and parents reported pain,^12^ and interviewing physicians at EDs have also shown that they want more knowledge on pain assessment.^26^ Children may benefit positively when health care professionals are educated in pain management, and especially in using nonpharmacological methods.^21^, ^27^, ^28^ This underscores the importance of the extra questions in this survey about distraction and positioning of the child during a procedure. This study did not ask specifically about the use of a pain assessment scale when managing pediatric pain, but a feasibility study has shown how a single observational pain assessment scale for children younger than 8 years could be easily used by both physicians and nurses to assess pain in children and adolescents and help to treat children's pain.^29^ The evidence behind the use of pain assessment scales and the outcome of care in hospitalized children is though still sparse.^30^

Vagnoli et al. (2019) showed that health care professionals in pediatric departments are more likely to acknowledge children feeling procedural pain compared with other departments. Our results supported these findings, as health care professionals in pediatric departments were more likely to trust the child's feeling of pain. There are, however, different areas where these health care professionals' might also need additional knowledge, as it has been shown that children experience not being consulted during medical procedures^31^ and that it is important to listen to the children's experiences.^13^, ^26^ The high turnover of employees at medical wards may have a negative effect on keeping a high standard for pediatric pain management. Future studies might investigate differences between health care professionals in more detail and determine how to integrate findings from this study when planning further education and interventions for health care professionals.

### 4.1 Strengths and limitations

The use of a reliable and valid survey is essential for the quality of the study. Several hospitals were included, and due to the large sample size, the study's validity is considered acceptable. The survey was sent out to a large number of health care professionals, and the risk of dropouts and a lower response rate were considered. The questionnaire consisted of 765 items, and 60% of the informants did not complete the survey and this may imply a risk of response bias. When contacted a second time, some replied that they could not spend so much time on a survey, especially when their primary work was not with children and adolescents. Held against the opportunity to compare all the health professionals, distributing the survey broadly was deemed valuable. Even though nurses, physicians, and MLTs were all included, it became clear in the early phase that the number of answers was not as high in the groups of physicians and MLTs as it was in the nurses. This should, however, also be seen in the light of the higher number of nurses and that children and adolescent pain management is a central part of the nursing profession and a highly discussed subject in Denmark. As the questionnaire was filled in anonymously, the same person could have answered the questionnaire more than once, even though they were asked not to.

Adding the “do not know” answer allowed respondents to answer more freely when feeling unsure about a statement, but it also led to a relatively large number of statements not being impossible to evaluate. This phenomenon was most prominent among the MLTs when asked about pharmaceutical statements, and they also mirrored their lack of knowledge about drug relieving pain management.

In this study, we were able to evaluate differences in knowledge and attitudes among health care professionals and departments. For some of the questions in the questionnaire, it will be possible to evaluate responses as more or less correct, and for others it will not be possible. Furthermore, we had to include new questions and add the possibility to answer “do not know” as we included different health care professionals. Therefore, the study is not able to distinguish between right answer and wrong answer. Further studies would be needed for that.

## 5 CONCLUSION

This study shows that knowledge and attitudes towards pain management in children varies among health care professionals in a hospital setting. Pain management is a complex multidisciplinary issue, and our study has identified areas for further alignment concerning key topics in the management of pediatric pain across disciplines and specialties, especially related to views on the care of children in pain, use of drugs, sociology, and psychology of pain, and the four mandatories. This study sets the stage for further education for all health care workers caring for hospitalized children.

## Supporting information


Appendix S1.
