# Supplementary material for: Pediatric pain knowledge and attitudes among health care professionals—A National Danish Survey
Source: Paediatr Neonatal Pain. 2023 Apr 10;5(3):76–85. doi: 10.1002/pne2.12104 (PMC10514779; doi:10.1002/pne2.12104)
Supplement: Supplementary file 1 — Appendix S1. [file PNE2-5-76-s001.docx]

**Supplementary file**

| **Statement** | **Profession (n) Mean (SD)** | **P-value** |
| --- | --- | --- |
| A1) Children tolerate pain better than adults do | Physician (202) 4.48 (0.910)  Nurse (457) 4.49 (0.976)  MLT (65) 3.98 (1.166) | Physician <> nurse 0.837  Physician <> MLT **0.000**  Nurse <> MLT **0.000** |
| A3) It is OK to carry out minor procedures, such as taking blood, without the use of analgesic drugs | Physician (202) 3.51 (1.450)  Nurse (462) 4.06 (1.305)  MLT (71) 2.39 (1.419) | Physician <> nurse **0.000**  Physician <> MLT **0.000**  Nurse <> MLT **0.000** |
| A4) Children under 2 years of age feel less pain than older children in similar situations | Physician (195) 4.80 (0.693)  Nurse (438) 4.74 (0.741)  MLT (55) 4.02 (1.381) | Physician <> nurse 0.406  Physician <> MLT **0.000**  Nurse <> MLT **0.000** |
| A5) A child who is crying and who says they are experiencing pain is likely to be in pain | Physician (202) 1.57 (0.731)  Nurse (453) 1.44 (0.706)  MLT 66) 1.88 (1.103) | Physician <> nurse **0.040**  Physician <> MLT **0.004**  Nurse <> MLT **0.000** |
| A6) Infants who are less than a month old may be intubated without pain medication | Physician (163) 4.86 (0.520)  Nurse (358) 4.82 (0.682)  MLT (16) 4.13 (1.310) | Physician <> nurse 0.595  Physician <> MLT **0.000**  Nurse <> MLT **0.000** |
| A7) Parents should be involved in the management of their child’s fever | Physician (203) 1.10 (0.321)  Nurse (459) 1.12 (0.408)  MLT (66) 1.39 (0.721) | Physician <> nurse 0.654  Physician <> MLT **0.000**  Nurse <> MLT **0.000** |
| A8) When a child can talk, they should be asked to rate their own pain intensity | Physician (200) 1.83 (0.952)  Nurse (447) 2.07 (1.181)  MLT (61) 2.30 (1.145) | Physician <> Nurse **0.011**  Physician <> MLT **0.004**  Nurse <> MLT 0.140 |
| A10) Postoperative pain in children should be eliminated | Physician (199) 1.02 (0.122)  Nurse (448) 1.01 (0.133)  MLT (49) 1.37 (0.602) | Physician <> nurse 0.923  Physician <> MLT **0.000**  Nurse <> MLT **0.000** |
| A11) When managing chronic pain in children the main goal of treatment is to control the pain | Physician (186) 1.79 (0.961)  Nurse (380) 1.82 (1.118)  MLT (42) 1.48 (0.671) | Physician <> nurse 0.744  Physician <> MLT 0.080  Nurse <> MLT **0.044** |
| A12) Parents exaggerate their child’s pain | Physician (195) 3.51 (1.132)  Nurse (425) 3.96 (1.129)  MLT (57) 2.74 (1.061) | Physician <> nurse **0.000**  Physician <> MLT **0.000**  Nurse <> MLT **0.000** |
| A13) Children do not need analgesic drugs before having burn dressings changed | Physician (178) 4.80 (0.602)  Nurse (400) 4.89 (0.491)  MLT (31) 4.58 (1.057) | Physician <> nurse 0.084  Physician <> MLT **0.046**  Nurse <> MLT **0.004** |
| A14) Infants who are less than a month old can be intubated without sedation | Physician (157) 4.83 (0.564)  Nurse (320) 4.84 (0.604)  MLT (13) 3.85 (1.573) | Physician <> nurse 0.881  Physician <> MLT **0.000**  Nurse <> MLT **0.000** |
| A15) School-aged children should only be given analgesic drugs if they ask for them | Physician (194) 3.99 (1.236)  Nurse (425) 4.24 (1.160)  MLT (53) 3.11 (1.476) | Physician <> nurse **0.021**  Physician <> MLT **0.000**  Nurse <> MLT **0.000** |
| A16) Procedural pain should be eliminated | Physician (196) 1.21 (0.491)  Nurse (446) 1.20 (0.493)  MLT (53) 1.81 (0.942) | Physician <> nurse 0.681  Physician <> MLT **0.000**  Nurse <> MLT **0.000** |
| A17) The level of pain suffered by a child can be established by giving him placebo medication | Physician (148) 4.35 (1.068)  Nurse (317) 4.40 (1.000)  MLT (24) 2.83 (1.090) | Physician <> nurse 0.632  Physician <> MLT **0.000**  Nurse <> MLT **0.000** |
| A20) Pain is to be expected if a child is in hospital | Physician (192) 3.62 (1.329)  Nurse (436) 3.67 (1.358)  MLT (57) 2.89 (1.423) | Physician <> nurse 0.643  Physician <> MLT **0.000**  Nurse <> MLT **0.000** |
| D6) The side effects of non-steroidal anti-inflammatory drugs (NSAIDs) e.g. diclofenac or ibuprofen, only occur when the drug is given orally | Physician (162) 4.78 (0.675)  Nurse (306) 4.58 (1.047)  MLT (6) 4.00 (1.095) | Physician <> nurse **0.027**  Physician <> MLT **0.042**  Nurse <> MLT 0.381 |
| D12) Non-steroidal anti-inflammatory drugs (NSAIDs) can irritate children’s digestive systems. | Physician (168) 1.23 (0.616)  Nurse (332) 1.45 (0.815)  MLT (14) 1.79 (0.802) | Physician <> nurse **0.002**  Physician <> MLT **0.008**  Nurse <> MLT 0.099 |
| D13) The risk of respiratory depression in children following the administration of opioids is no more likely than in adults, provided that the correct dosage is given | Physician (149) 1.50 (1.044)  Nurse (295) 1.78 (1.338)  MLT (3) 2.67 (0.577) | Physician <> nurse **0.027**  Physician <> MLT 0.109  Nurse <> MLT 0.219 |
| D24) Non-steroidal anti-inflammatory drugs (NSAID) and opioid medication given together provide better analgesic | Physician (155) 1.34 (0.723)  Nurse (285) 1.56 (0.939)  MLT (4) 2.00 (1.155) | Physician <> nurse **0.011**  Physician <> MLT 0.131  Nurse <> MLT 0.312 |
| E1) The environment in which children grow up has a major influence on the way they express pain | Physician (168) 1.38 (0.556)  Nurse (355) 1.73 (0.930)  MLT (42) 1.98 (0.869) | Physician <> nurse **0.000**  Physician <> MLT **0.000**  Nurse <> MLT 0.072 |
| E2) School-aged children cannot learn how to use a patient-controlled analgesia pump | Physician (139) 4.66 (0.757)  Nurse (272) 4.54 (0.854)  MLT (42) 4.00 (0.926) | Physician <> nurse 0.168  Physician <> MLT **0.003**  Nurse <> MLT **0.013** |
| E3) A child’s cultural background affects the way they experience pain | Physician (167) 1.77 (1.130)  Nurse (358) 1.99 (1.199  MLT (42) 2.14 (1.160) | Physician <> nurse **0.042**  Physician <> MLT 0.065  Nurse <> MLT 0.432 |
| E4) The way that children express pain is affected by their temperament | Physician (172) 1.35 (0.589)  Nurse (360) 1.65 (0.835)  MLT (50) 1.70 (0.789) | Physician <> nurse **0.000**  Physician <> MLT **0.006**  Nurse <> MLT 0.653 |
| E5) Changes in a child’s behavior can be used to assess their pain | Physician (173) 1.47 (0.736)  Nurse (370) 1.52 (0.726)  MLT (37) 1.89 (0.614) | Physician <> nurse 0.474  Physician <> MLT **0.001**  Nurse <> MLT **0.003** |
| E6) It is difficult to make a distinction between pain and fear in children | Physician (173) 1.87 (0.849)  Nurse (370) 2.18 (1.030)  MLT (50) 2.30 (1.093) | Physician <> nurse **0.001**  Physician <> MLT **0.006**  Nurse <> MLT 0.399 |
| E7) Chronic pain in children does not usually cause mood changes | Physician (134) 4.69 (0.616)  Nurse (251) 4.48 (0.826)  MLT (19) 3.53 (1.349) | Physician <> nurse **0.012**  Physician <> MLT **0.000**  Nurse <> MLT **0.000** |
| E8) When assessing a child’s pain, it is important to first ascertain their stage of cognitive development | Physician (148) 2.57 (1.246)  Nurse (322) 3.09 (1.468)  MLT (20) 2.50 (1.000) | Physician <> nurse **0.000**  Physician <> MLT 0.839  Nurse <> MLT 0.068 |
| E9) The way children experience pain is influenced by their parents’ behavior | Physician (175) 1.31 (0.566)  Nurse (374) 1.55 (0.719)  MLT (58) 1.40 (0.566) | Physician <> nurse **0.000**  Physician <> MLT 0.425  Nurse <> MLT 0.114 |
| E10) Letting a child know what to expect before a painful procedure will mean that the child experiences less pain than a child who has not been given this information | Physician (167) 1.58 (0.747)  Nurse (365) 1.61 (0.843)  MLT (53) 1.40 (0.560) | Physician <> nurse 0.696  Physician <> MLT **0.006**  Nurse <> MLT **0.006** |
| E11: Children can sleep even if they are in pain | Physician (166) 1.99 (1.101)  Nurse (350) 2.31 (1.296)  MLT (37) 2.32 (1.107) | Physician <> nurse **0.006**  Physician <> MLT 0.133  Nurse <> MLT 0.930 |
| E12) children between the ages six to twelve months will have no lasting memories of painful procedures | Physician (131) 4.02 (1.301)  Nurse (281) 4.22 (1.135)  MLT (37) 2.61 (1.407) | Physician <> nurse 0.102  Physician <> MLT **0.000**  Nurse <> MLT **0.000** |

| **Statement** | **Workplace (n)** | **Mean (SD)** | **P-value** |
| --- | --- | --- | --- |
| A1) Children tolerate pain better than adults do | Pediatric (359)  Anesthesia (168)  ER (65)  MLT (72)  Surgery (88) | 4.48 (0.985)  4.67 (0.740)  4.29 (1.112)  4.06 (1.112)  4.20 (1.095) | **0.046**  0.144  **0.001**  **0.016** |
| A3) It is OK to carry out minor procedures, such as taking blood, without the use of analgesic drugs | Pediatric (358)  Anesthesia (173)  ER (65)  MLT (78)  Surgery (89) | 4.06 (1.309)  3.97 (1.325)  3.49 (1.480)  2.42 (1.437)  3.30 (1.480) | 0.459  **0.002**  **0.000**  **0.000** |
| A4) Children under 2 years of age feel less pain than older children in similar situations | Pediatric (359)  Anesthesia (168)  ER (65)  MLT (72)  Surgery (88) | 4.84 (0.586)  4.74 (0.782)  4.49 (0.782)  4.03 (1.390)  4.61 (0.864) | 0.197  **0.003**  **0.000**  **0.026** |
| A5) A child who is crying and who says they are experiencing pain is likely to be in pain | Pediatric (354)  Anesthesia (171)  ER (64)  MLT (73)  Surgery (87) | 1.47 (0.703)  1.44 (0.729)  1.44 (0.753)  1.93 (0.677)  1.56 (0.677) | 0.670  0.719  **0.000**  0.329 |
| A6) Infants who are less than a month old may be intubated without pain medication | Pediatric (304)  Anesthesia (144)  ER (43)  MLT (22)  Surgery (48) | 4.86 (0.592)  4.80 (0.705)  4.79 (0.742)  3.86 (1.457)  4.71 (0.713) | 0.369  0.530  **0.000**  0.156 |
| A7) Parents should be involved in the management of their child’s fever | Pediatric (358)  Anesthesia (171)  ER (64)  MLT (73)  Surgery (90) | 1.07 (0.250)  1.19 (0.564)  1.16 (0.366)  1.37 (0.697)  1.17 (0.480) | **0.003**  0.129  **0.000**  0.051 |
| A8) When a child can talk, they should be asked to rate their own pain intensity | Pediatric (350)  Anesthesia (171)  ER (62)  MLT (66)  Surgery (85) | 1.99 (1.118)  1.82 (1.054)  2.16 (1.244)  2.35 (1.130)  2.13 (1.055) | 0.101  0.275  **0.018**  0.314 |
| A10) Postoperative pain in children should be eliminated | Pediatric (352)  Anesthesia (169)  ER (58)  MLT (55)  Surgery (89) | - 1. (0.075)   1.02 (0.132)  1.07 (0.317)  1.29 (0.533)  1.04 (0.257) | 0.540  **0.034**  **0.000**  0.116 |
| A12) Parents exaggerate their child’s pain | Pediatric (337)  Anesthesia (160)  ER (60)  MLT (629  Surgery (83) | 3.90 (1.098)  4.05 (1.045)  3.47 (1.295)  2.79 (1.073)  3.28 (1.180) | 0.153  **0.006**  **0.000**  **0.000** |
| A13) Children do not need analgesic drugs before having burn dressings changed | Pediatric (315)  Anesthesia (146)  ER (58)  MLT (37)  Surgery (79) | 4.92 (0.429)  4.88 (0.505)  4.71 (0.817)  4.68 (0.818)  4.63 (0.787) | 0.553  **0.010**  **0.015**  **0.000** |
| A14) Infants who are less than a month old can be intubated without sedation | Pediatric (277)  Anesthesia (136)  ER (32)  MLT (17)  Surgery (46) | 4.84 (0.589)  4.85 (0.615)  4.94 (0.354)  3.88 (1.453)  4.65 (0.737) | 0.820  0.406  **0.000**  0.071 |
| A15) School-aged children should only be given analgesic drugs if they ask for them | Pediatric (336)  Anesthesia (162)  ER (58)  MLT (59)  Surgery (81) | 4.25 (1.141)  4.15 (1.170)  4.05 (1.317)  3.14 (1.432)  3.96 (1.259) | 0.391  0.254  **0.000**  0.057 |
| A16) Procedural pain should be eliminated | Pediatric (348)  Anesthesia (168)  ER (58)  MLT (60)  Surgery (86) | 1.19 (0.502)  1.19 (0.464)  1.29 (0.562)  1.85 (0.954)  1.22 (0.445) | 0.987  0.181  **0.000**  0.633 |
| A17) The level of pain suffered by a child can be established by giving him placebo medication | Pediatric (250)  Anesthesia (127)  ER (45)  MLT (28)  Surgery (57) | 4.36 (1.033)  4.54 (0.889)  4.20 (1.140)  2.96 (1.170)  4.18 (1.151) | 0.119  0.338  **0.000**  0.223 |
| A20) Pain is to be expected if a child is in hospital | Pediatric (339)  Anesthesia (167)  ER (58)  MLT (63)  Surgery (82) | 3.65 (1.355)  3.74 (1.286)  3.90 (1.320)  2.84 (1.439)  3.34 (1.407) | 0.508  0.203  **0.000**  0.062 |
| A21) Parents should be prepared for their role in supporting their child during a painful procedure | Pediatric (339)  Anesthesia (167)  ER (58)  MLT (63)  Surgery (82) | - 1. (0.240)   1.14 (0.528)  1.05 (0.222)  1.10 (0.345)  1.09 (0.328) | **0.000**  0.571  0.095  0.098 |
| D1) In treating pain in children only one class of analgesic drug should be used at a time | Pediatric (293)  Anesthesia (138)  ER (47)  MLT (17)  Surgery (60) | 4.65 (0.833)  4.67 (0.849)  4.15 (1.251)  2.47 (1.068)  4.20 (1.388) | 0.854  **0.001**  **0.000**  **0.001** |
| D6) The side effects of non-steroidal anti-inflammatory drugs (NSAIDs) e.g. diclofenac or ibuprofen, only occur when the drug is given orally | Pediatric (244)  Anesthesia (136)  ER (35)  MLT (9)  Surgery (63) | 4.70 (0.838)  4.71 (0.842)  4.20 (1.324)  4.11 (1.054)  4.51 (1.091) | 0.933  **0.003**  0.058  0.131 |
| D8) Non-steroidal anti-inflammatory drugs (NSAIDs) are unsuitable for use with some children who have asthma | Pediatric (148)  Anesthesia (89)  ER (22)  MLT (4)  Surgery (32) | 3.53 (1.584)  2.57 (1.559)  2.77 (1.510)  3.50 (1.000)  1.94 (1.243) | **0.000**  **0.031**  0.965  **0.000** |
| D10) Paracetamol (acetaminophen) is used for treatment of mild pain | Pediatric (316)  Anesthesia (153)  ER (48)  MLT (45)  Surgery (78) | 1.16 (0.545)  1.13 (0.511)  1.25 (0.668)  1.49 (0.661)  1.15 (0.626) | 0.555  0.330  **0.000**  0.881 |
| D13) The risk of respiratory depression in children following the administration of opioids is no more likely than in adults, provided that the correct dosage is given | Pediatric (233)  Anesthesia (139)  ER (38)  MLT (4)  Surgery (44) | 1.68 (1.250)  1.55 (1.229)  1.79 (1.044)  2.75 (0.500)  2.09 (1.444) | 0.310  0.623  0.090  **0.046** |
| D14) The most common reason for the increased need for analgesic drugs in the treatment of children with cancer is the child’s increasing tolerance to the drugs | Pediatric (132)  Anesthesia (87)  ER (19)  MLT (5)  Surgery (35) | 2.35 (1.325)  1.84 (0.901)  2.32 (1.293)  2.40 (0.548)  2.49 (1.522) | **0.003**  0.913  0.927  0.556 |
| D15) Opioid medication given to manage chronic pain in children should be given on a regular basis | Pediatric (192)  Anesthesia (113)  ER (23)  MLT (5)  Surgery (36) | 2.21 (1.372)  1.47 (0.825)  2.13 (1.359)  2.80 (1.095)  1.81 (1.167) | **0.000**  0.770  0.279  0.067 |
| D16) The use of sedative drugs is an effective way of eliminating pain in children | Pediatric (253)  Anesthesia (137)  ER (33)  MLT (4)  Surgery (43) | 4.42 (1.022)  4.66 (0.701)  3.97 (1.185)  3.25 (1.258)  4.07 (1.078) | **0.018**  **0.013**  **0.016**  **0.030** |
| D17) Postoperatively children should not be given analgesic drugs until they ask for them | Pediatric (306)  Anesthesia (151)  ER (43)  MLT (19)  Surgery (73) | 4.95 (0.251)  4.82 (0.578)  4.72 (0.630)  4.00 (1.054)  4.70 (0.758) | **0.011**  **0.006**  **0.000**  **0.000** |
| D18) Non-steroidal anti-inflammatory drugs (NSAIDs) and opioids cannot be given at the same time | Pediatric (247)  Anesthesia (141)  ER (36)  MLT (2)  Surgery (61) | 4.73 (0.777)  4.92 (0.464)  4.28 (1.186)  3.00 (0.000)  4.62 (0.969) | **0.020**  **0.001**  **0.002**  0.317 |
| D19) Respiratory depression is rare in children who are receiving long -term opioid analgesic | Pediatric (171)  Anesthesia (103)  ER (18)  MLT (2)  Surgery (31) | 1.98 (1.241)  1.64 (0.979)  2.72 (1.179)  3.00 (0.000)  2.42 (1.259) | **0.021**  **0.010**  0.216  0.051 |
| D20) Non-steroidal anti-inflammatory drugs (NSAIDs) increase the adverse respiratory effect of opioids | Pediatric (137)  Anesthesia (113)  ER (24)  MLT (2)  Surgery (26) | 4.60 (0.861)  4.66 (0.830)  4.13 (1.191)  3.00 (0.000)  4.19 (1.234) | 0.576  **0.020**  **0.015**  **0.039** |
| D21) Paracetamol (acetaminophen) is unsuitable for children who have asthma | Pediatric (258)  Anesthesia (131)  ER (36)  MLT (4)  Surgery (55) | 4.93 (0.341)  4.95 (0.379)  4.78 (0.591)  4.25 (0.957)  4.73 (0.827) | 0.800  0.056  **0.003**  **0.002** |
| D23) There is an antagonist (antidote) to the adverse effect of respiratory depression caused by opioids | Pediatric (275)  Anesthesia (143)  ER (43)  MLT (4)  Surgery (58) | 1.12 (0.492)  1.13 (0.674)  1.14 (0.413)  2.75 (0.500)  1.12 (0.595) | 0.773  0.799  **0.000**  0.957 |
| D24) Non-steroidal anti-inflammatory drugs (NSAID) and opioid medication given together provide better analgesic | Pediatric (213)  Anesthesia (143)  ER (36)  MLT (5)  Surgery (58) | 1.62 (1.019)  1.27 (0.663)  1.67 (0.663)  2.20 (1.095)  1.36 (0.641) | **0.000**  0.762  0.137  **0.044** |
| D25) Regional (e.g. epidural or caudal infiltration) anesthesia is a good method of pain management after surgery | Pediatric (206)  Anesthesia (143)  ER (31)  MLT (6)  Surgery (55) | 1.57 (1.018)  1.34 (0.759)  1.55 (0.850)  2.50 (1.643)  1.84 (1.259) | **0.026**  0.897  **0.022**  0.076 |
| D26) Paracetamol and an opioid cannot be given at the same time | Pediatric (283)  Anesthesia (145)  ER (43)  MLT (4)  Surgery (61) | 4.89 (0.441)  4.97 (0.342)  4.86 (0.467)  3.50 (1.000)  4.92 (0.378) | 0.094  0.624  **0.000**  0.683 |
| E1) The environment in which children grow up has a major influence on the way they express pain | Pediatric (294)  Anesthesia (134)  ER (45)  MLT (45)  Surgery (63) | 1.63 (0.819)  1.59 (0.860)  1.73 (0.939)  1.96 (0.928)  1.54 (0.877) | 0.628  0.461  **0.018**  0.433 |
| E2) School-aged children cannot learn how to use a patient-controlled analgesia pump | Pediatric (220)  Anesthesia (131)  ER (33)  MLT (17)  Surgery (40) | 4.55 (0.856)  4.77 (0.602)  4.42 (0.708)  4.12 (0.928)  4.38 (1.148) | **0.012**  0.425  **0.037**  0.224 |
| E3) A child’s cultural background affects the way they experience pain | Pediatric (295)  Anesthesia (133)  ER (46)  MLT (45)  Surgery (65) | 2.03 (1.267)  1.76 (1.016)  1.98 (1.256)  2.24 (1.209)  1.69 (0.967) | **0.030**  0.794  0.249  **0.038** |
| E4) The way that children express pain is affected by their temperament | Pediatric (300)  Anesthesia (135)  ER (47)  MLT (52)  Surgery (65) | 1.61 (0.852)  1.44 (0.594)  1.68 (0.862)  1.69 (0.781)  1.43 (0.728) | **0.030**  0.582  0.502  0.089 |
| E5) Changes in a child’s behavior can be used to assess their pain | Pediatric (306)  Anesthesia (143)  ER (44)  MLT (40)  Surgery (64) | 1.48 (0.697)  1.56 (0.784)  1.57 (0.846)  1.85 (0.622)  1.42 (0.708) | 0.284  0.454  **0.003**  0.559 |
| E7) Chronic pain in children does not usually cause mood changes | Pediatric (237)  Anesthesia (95)  ER (28)  MLT (21)  Surgery (40) | 4.55 (0.772)  4.67 (0.675)  4.36 (0.911)  3.38 (1.396)  4.50 (0.750) | 0.199  0.233  **0.000**  0.723 |
| E10) Letting a child know what to expect before a painful procedure will mean that the child experiences less pain than a child who has not been given this information | Pediatric (300)  Anesthesia (139)  ER (47)  MLT (58)  Surgery (61) | 1.58 (0.812)  1.51 (0.685)  1.83 (1.090)  2.07 (1.006)  1.75 (0.767) | 0.415  0.054  **0.000**  0.134 |
| E11: Children can sleep even if they are in pain | Pediatric (296)  Anesthesia (133)  ER (44)  MLT (41)  Surgery (58) | 2.12 (1.206)  2.41 (1.314)  2.25 (1.314)  2.59 (1.224)  2.26 (1.278) | **0.028**  0.514  **0.025**  0.434 |
| E12) Children between the ages six to twelve months will have no lasting memories of painful procedures | Pediatric (263)  Anesthesia (98)  ER (27)  MLT (35)  Surgery (35) | 4.24 (1.132)  4.30 (1.168)  3.70 (1.103)  2.71 (1.459)  4.05 (1.267) | 0.690  **0.027**  **0.000**  **0.003** |
| FA9) Re-scoring pain is an important measurement when to assess the effect of the pain treatment | Pediatric (358)  Anesthesia (172)  ER (64)  MLT (42)  Surgery (88) | 1.11 (0.375)  1.14 (0.364)  1.31 (0.639)  1.52 (0.773)  1.27 (0.582) | 0.559  **0.002**  **0.000**  **0.004** |
| FA18) When having a procedure done it is evidently that helping a child coping with pain and anxiety can be done by four actions: Emla, position, sugar water/breastfeeding and distraction | Pediatric (326)  Anesthesia (148)  ER (49)  MLT (67)  Surgery (58) | 1.19 (0.511)  1.28 (0.650)  1.35 (0.597)  1.31 (0.556)  1.62 (0.875) | 0.126  0.093  0.133  **0.000** |
| FA19) Restraining small children may have consequences many years ahead | Pediatric (328)  Anesthesia (157)  ER (55)  MLT (66)  Surgery (79) | 1.39 (0.734)  1.24 (0.521)  1.51 (0.998)  2.15 (1.206)  1.68 (0.955) | **0.048**  0.310  **0.000**  **0.004** |
| FA22) There may be situations (apart from life threatening) where it is best to get a procedure done fast by restraining the child | Pediatric (335)  Anesthesia (164)  ER (57)  MLT (70)  Surgery (83) | 3.10 (1.490)  3.66 (1.476)  2.75 (1.596)  1.87 (1.048)  2.78 (1.531) | **0.000**  0.095  **0.000**  0.074 |
| FC2) The best place for a child to be during a stitching procedure is to sit with a parent | Pediatric (323)  Anesthesia (156)  ER (54)  MLT (69)  Surgery (78) | 1.38 (0.726)  1.62 (0.731)  1.63 (0.808)  2.54 (1.431)  1.42 (0.614) | **0.003**  **0.037**  **0.000**  0.662 |
| FC7) It is best and fastest to do a blood sample while the child is lying on the bed | Pediatric (311)  Anesthesia (130)  ER (52)  MLT (63)  Surgery (76) | 4.38 (1.034)  4.15 (1.096)  4.13 (1.104)  2.57 (1.340)  3.93 (1.052) | **0.045**  0.128  **0.000**  **0.004** |
| FC12) If at mother is nervous and scared when her 4-year-old child has to have a procedure done, it is better if the staff takes over and sends out the parents | Pediatric (312)  Anesthesia (154)  ER (52)  MLT (63)  Surgery (76) | 4.26 (1.085)  4.03 (1.234)  4.12 (1.323)  2.67 (1.391)  3.61 (1.212) | **0.049**  0.407  **0.000**  **0.000** |
| FD2) Local anesthesia (Emla) can be used by all children, even neonate | Pediatric (260)  Anesthesia (104)  ER (33)  MLT (43)  Surgery (46) | 2.93 (1.784)  2.12 (1.578)  2.33 (1.555)  3.30 (1.626)  1.50 (1.070) | **0.000**  0.052  0.174  **0.000** |
| FD4) By the neonate child it is less harmful to do a venous blood sample than a capillary | Pediatric (181)  Anesthesia (42)  ER (13)  MLT (50)  Surgery (15) | 3.90 (1.447)  4.38 (0.936)  3.92 (1.256)  3.68 (1.301)  3.67 (1.447) | **0.037**  0.943  0.322  0.531 |
| FD7) When using Emla one can assure the child that the procedure won’t hurt | Pediatric (308)  Anesthesia (152)  ER (50)  MLT (67)  Surgery (76) | 4.46 (0.990)  4.06 (1.298)  4.24 (1.153)  3.30 (1.487)  4.18 (1.140) | **0.000**  0.203  **0.000**  0.059 |
| FD9) Relieving pain during a stitching procedure, children in the age of 0-1 year, should be given the opportunity to breastfeed or sugar water | Pediatric (315)  Anesthesia (140)  ER (42)  MLT (64)  Surgery (60) | 1.53 (1.010)  1.66 (1.064)  1.64 (1.100)  1.95 (1.161)  1.78 (0.940) | 0.215  0.521  **0.003**  0.088 |
| FD22) Local anesthetic crème is great when doing a heel blood sample | Pediatric (191)  Anesthesia (60)  ER (22)  MLT (38)  Surgery (26) | 3.63 (1.626)  2.35 (1.471)  2.50 (1.439)  4.29 (1.271)  2.27 (1.373) | **0.000**  **0.001**  **0.017**  **0.000** |
| FD27) An infant child should not breastfeed during a harmful procedure, as it may destroy an important bond between mother and child | Pediatric (301)  Anesthesia (105)  ER (37)  MLT (53)  Surgery (42) | 4.73 (0.828)  4.48 (1.048)  4.22 (1.084)  4.15 (1.026)  4.12 (1.435) | **0.023**  **0.003**  **0.000**  **0.000** |
